# A dual-chamber leadless pacemaker implantation across percutaneous tricuspid valve-in-valve prosthesis in a patient with Ebstein anomaly

**DOI:** 10.1016/j.hrcr.2024.10.016

**Published:** 2024-10-18

**Authors:** Bandar Saeed Al-Ghamdi, Maher Dhabi Salem, Mohammed Najmeddine Echahidi

**Affiliations:** Adult Cardiology Section, Heart Centre Department, Alfaisal University, Riyadh, Saudi Arabia

**Keywords:** Ebstein anomaly, Tricuspid valve prosthesis, Leadless pacemaker, Heart block, Dual chamber

**Key Teaching Points**

• Ebstein anomaly of the tricuspid valve is frequently associated with tachyarrhythmias and bradycardias.
• The optimal approach for cardiac implantable electronic device implantation and management in patients with Ebstein anomaly remains to be determined.
• Implanting a leadless pacemaker device, single or dual chamber, in patients with Ebstein anomaly with significant tricuspid valve regurgitation or tricuspid valve prostheses is an attractive option.

## Introduction

Ebstein anomaly of the tricuspid valve (TV) is frequently associated with tachyarrhythmias and bradycardias.^1^^,^^2^ Therefore, patients with Ebstein anomaly may require a cardiac implantable electronic device, such as a pacemaker for bradycardia or an implantable cardioverter-defibrillator (ICD) for tachyarrhythmia management. The optimal approach for cardiac implantable electronic device implantation and management in these patients remains to be determined.^3^

We present a case of a dual-chamber leadless pacemaker system implantation (Aveir DR; Abbott) in a patient with Ebstein anomaly and severe TV regurgitation (TR) who had multiple TV interventions, including a valve-in-valve procedure and complete heart block with failing epicardial right ventricular (RV) pacing lead.

This approach may enhance the pacing management options in this subgroup of patients with congenital heart disease.

## Case report

A 51-year-old woman had an Ebstein anomaly with severe TR and atrial septal defect. She had no palpitations and no documented arrhythmia. She underwent TV replacement using a Carpentier-Edwards bioprosthesis (size 25 mm; Edwards Lifesciences) and atrial septal defect closure in 1983. She had TV bioprosthesis degeneration with severe stenosis and moderate regurgitation, so she underwent a redo TV replacement using a St. Jude tissue valve bioprosthesis (size 33 mm; St. Jude Medical). The coronary sinus (CS) was found to be draining anomalously into the RV, and it was brought back to drain into the right atrium (RA) in October 2009. Postoperatively, she had a complete heart block and, subsequently, underwent a permanent bipolar epicardial pacemaker system implantation 2 weeks later. She was pacemaker-dependent during follow-up and underwent a pacemaker generator change in 2018. She developed TV bioprosthesis degeneration with moderate to severe TR and subsequently underwent percutaneous tricuspid valve-in-valve implantation using an Edwards valve bioprosthesis (size 29 mm) in 2023. She was noted to have a progressively increasing RV pacing threshold and rapid pacemaker battery depletion.

Electrocardiogram showed a ventricular-paced rhythm at 60 beats/min.

The echocardiogram showed normal left ventricle size and systolic function with a left ventricular ejection fraction >55%. The RV was moderately dilated with impaired longitudinal contraction. Both atria were mildly dilated, and atrial septal defect patch closure with no residual shunt was noted. The TV valve-in-valve bioprosthesis was well-seated with mild intravalvular regurgitation. The pressure gradients across the valve were normal (mean gradient, 4.3 mm Hg at 60 beats/min).

The option of a new epicardial RV lead insertion with pacemaker generator change was felt to be invasive and challenging because of the previous cardiac surgeries. CS lead placement was considered suboptimal in this patient due to her CS anatomy, with an earlier surgical redirection of CS to RA and the questionability of lead stability in a pacemaker-dependent patient. The option of a dual-chamber transvenous pacemaker implantation with the limitation of RV lead crossing the bioprosthetic TV versus a dual-chamber leadless pacemaker implantation was considered. After a shared decision-making with the patient, we chose the leadless pacemaker option.

### Implantation procedure

**Figure 1:**
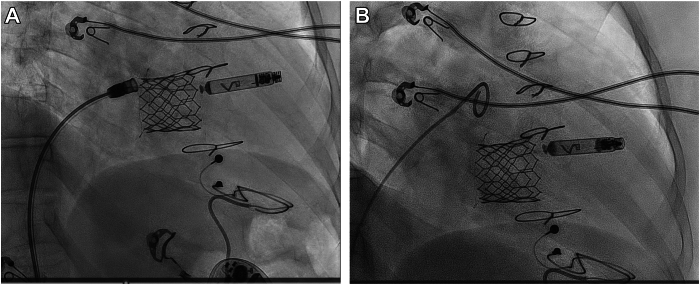
The Aveir VR delivery system (Abbott) with the device released from the catheter while in tethered mode (**A**) and after the tether cables were released from the docking button (**B**) in the right anterior oblique 30-degree projection.

The procedure was performed under conscious sedation and local anesthesia in the electrophysiology laboratory. Venous access was obtained in the right femoral vein, and a 9F short sheath was inserted. The Aveir VR (Abbott) 27F introducer sheath was inserted after the entry-site dilatation. Then, the Aveir delivery catheter with the Aveir VR pacemaker was introduced into the RA under fluoroscopy guidance higher than the level we usually aim for with this system to avoid the pouch below the TV prosthesis. A counterclockwise torque was applied to allow the crossing of the TV prosthesis. The bioprosthetic valve was crossed in the right anterior oblique projection view. Then, clockwise torque was applied to have a septal position, confirmed by left anterior oblique projection. The best position was found at the mid-interventricular septum without contact with the TV prosthesis. The protective sleeve was slowly withdrawn while the device was gently advanced to have good contact with the ventricular myocardium. The device was fixated to the myocardium with 1.5 clockwise rotations as evaluated by the radiopaque chevron maker on the device's body. Once fixated, the device was released from the catheter, while remaining in the tethered mode. Acceptable current of injury and suitable pacing parameters were achieved from the first attempt with an R-wave amplitude of 6.0 mV, impedance of 550 Ω, and pacing threshold of 0.75 V at 0.4 milliseconds. The device was released, and the delivery system was withdrawn from the delivery sheath (Figure 1).

**Figure 2:**
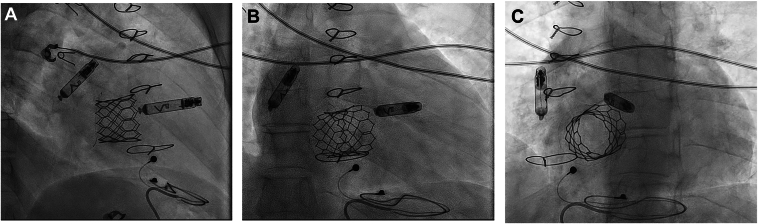
The 2 devices position in the right anterior oblique 30 degrees (**A**), anteroposterior (**B**), and left anterior oblique 30 degrees (**C**) projections.

An upper RA angiogram with an injection of 10 mL of contrast via a pigtail catheter showed a good position at the base of the right atrial appendage remnant. Then, the Aveir delivery catheter with the Aveir AR pacemaker (Abbott) was introduced to the RA. Deployment of the Aveir AR at this site was successful. However, the P-wave amplitude was suboptimal. The system was repositioned laterally with good parameters (P wave of 1.9 mV, impedance of 370 Ω, and pacing threshold of 1.0 V at 0.4 milliseconds). The stability test showed the device in an acceptable position. The device was released, the delivery system was withdrawn from the delivery sheath, the introducer sheath was removed, and figure-of-8 stitche was placed. The position of the 2 devices was optimal in fluoroscopy projections (Figure 2).

The device's communication was achieved with implant-to-implant (i2i; Abbott) communication technology with an excellent beat-to-beat atrioventricular (AV) synchrony.

**Figure 3:**
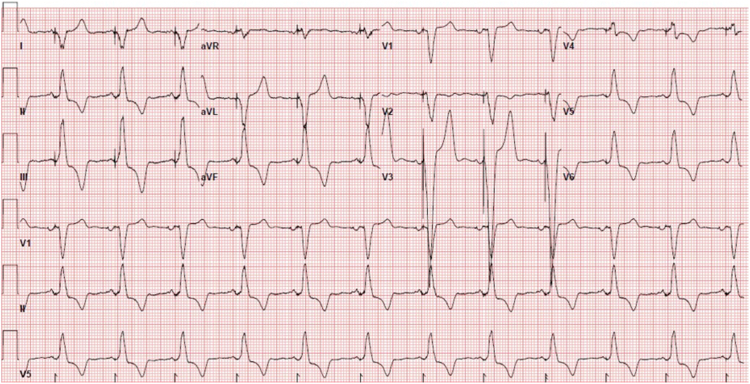
12-Lead electrocardiogram showing atrial sensed-ventricular paced rhythm.

A routine device checkup on the day after the procedure confirmed the excellent electrical parameters. The bedside transthoracic echocardiography showed no change in the tricuspid prosthesis structure and function, providing reassurance about the procedure's success. Electrocardiogram showed atrial sensed ventricular paced rhythm with good tracking of atrial activity (Figure 3).

## Discussion

Patients with Ebstein anomaly undergoing TV surgery who need pacing typically undergo epicardial lead implantation or externalization of transvenous leads to the TV to avoid lead placement across the valve leaflets.^3^ In a patient with Ebstein anomaly with an indication for pacing and tachycardia therapy, a combination of a leadless pacemaker and subcutaneous implantable cardioverter defibrillator implantation has been reported.^4^

Significant TR is typically managed with TV surgical repair or replacement or by the less invasive approach of percutaneous TV interventions with valve replacement, repair, or caval valves.^5^ Pacemaker implantation in patients with TV prostheses represents a challenge. TV mechanical prosthesis is considered an absolute contraindication for transvenous endocardial RV lead implantation.^6^ However, transvenous endocardia lead^7^ and leadless pacemaker implantation in patients with mechanical TV have been described in anecdotal case reports.^8^ Although inserting a transvenous RV lead across a TV bioprosthesis is feasible, it is considered a suboptimal solution because of the possible valve leaflet damage or valve apparatus dysfunction and the potential risk of infective endocarditis over time.^6^^,^^9^

Surgical placement of an epicardial lead is another option, but it has a higher procedure risk and a more extended recovery period. It may not be anatomically feasible in patients with prior cardiac surgery.^6^ Furthermore, epicardial leads are historically associated with a higher failure rate.^10^

LV-only pacing through the CS is another possible alternative. However, due to the limited lead stability, it may not be the preferred option in pacemaker-dependent patients.^11^^,^^12^

Leadless pacemakers are becoming an attractive alternative in patients with absolute or relative contraindications to standard endocardial pacemaker implants.^13^ Furthermore, leadless pacemakers do not significantly impact TR if the basal septum position is avoided.^14^ Recently, a dual-chamber leadless pacemaker system became available, consisting of 2 devices introduced percutaneously—1 in the RA and 1 in the RV—with wireless communication and synchronization between the 2 pacemakers. These devices provide AV synchrony and extend the leadless pacemaker therapy option for a broader range of indications.^15^

The currently available dual-chamber leadless pacemaker system (Aveir DR) achieved a 98.3% implant success rate and >97% successful AV synchrony, despite different underlying bradycardias.^15^ The system gained US Food and Drug Administration approval on July 5, 2023. However, the current leadless pacing systems lack antitachycardia pacing, which is a shortcoming, particularly in patients with congenital heart disease with high rates of atrial tachyarrhythmia.

Our patient was pacer-dependent and had epicardial RV pacing for years with a stable left ventricular ejection fraction. However, RV-pacing cardiomyopathy may occur in the future and may require upgrading to cardiac resynchronization therapy. Fortunately, the Aveir leadless pacemaker is a helix-based active fixation system with a dedicated retrieval catheter designed for acute and chronic retrieval attempts, so the system can be removed if needed.

To our knowledge, no published data exist on patients with Ebstein anomaly and transcatheter TV prostheses who underwent dual-chamber leadless pacemaker implantation. The TV's apical displacement in those patients may make leadless pacemaker implantation challenging, and proper device position on the septum needs to be assured.

## Conclusion

Leadless pacemakers present a promising option for patients with Ebstein anomaly and significant TV disease who need pacing. Dual-chamber leadless pacemaker implantation is feasible in these patients and helps maintain AV synchrony.

## Disclosures

The authors have no conflicts of interest to disclose.
